# Regarding Cardiac Disease Patterns in Northern Malawi: Epidemiologic Transition Perspective

**DOI:** 10.2188/jea.JE20090081

**Published:** 2009-09-05

**Authors:** Okechukwu S Ogah

**Affiliations:** Department of Medicine, Federal Medical Centre, Abeokuta, Ogun State, Nigeria

Dear Sir:

Kindly permit me to make a comment on this paper.

I read with great delight the paper written by Soliman and Juma^1^ titled “Cardiac Disease Patterns in Northern Malawi: Epidemiologic Transition Perspective” and published in J. Epidemiol 2008;18(5):204–208. I must thank and congratulate the authors for their good work and contribution to the clinical epidemiology of cardiac disease in Malawi.

However, in establishing the possible existence of epidemiologic transition in this country, the authors should have made a more determined effort to find similar previous work done in this country (research they claimed did not exist).

A MEDLINE search indicates that there were at least two previous studies—a clinical study by Brown^2^ and an echocardiographic study by Hakim.^3^ Conclusions that can be drawn from these studies include (see Table below):

1. The increase in the prevalence of hypertension,
2. The decrease or disappearance of syphilitic heart disease as a cause of cardiac morbidity in Malawi, possibly due to increased availability of antimicrobial agents, and
3. The gradual emergence of coronary heart disease.

**Table:** 

NO	Author	Year	No	HHDX (%)	VHDX (%)	DCM (%)	CP (%)	PDX (%)	SHDX (%)	IHD (%)	CHD (%)	Misc (%)
1	Soliman^1^	2008	3908	24.2	33.6	18.6	—	14.2	0	0.08	4.4	1.58
2	Hakim^3^	1998	1153	13.3	25.1	22.0	3.9	22.4	—	—	3.8	9.7
3	Brown^2^	1975	114	16	35	5	8	7	4.4	—	2.5	22.8

HHDX = Hypertensive heart disease, VHDX = valvular heart disease, DCM = Dilated Cardiomyopathy, CP = Cor pulmonale, PDX = Pericardial diseases, SHDX = Syphilitic heart disease, IHD = Ischemic Heart Disease, CHDX = Congenital heart disease, Misc = Miscellaneous
